# Seaweed intake and blood pressure levels in healthy pre-school Japanese children

**DOI:** 10.1186/1475-2891-10-83

**Published:** 2011-08-10

**Authors:** Keiko Wada, Kozue Nakamura, Yuya Tamai, Michiko Tsuji, Yukari Sahashi, Kaori Watanabe, Sakiko Ohtsuchi, Keiko Yamamoto, Kyoko Ando, Chisato Nagata

**Affiliations:** 1 Department of Epidemiology and Preventive Medicine, Gifu University Graduate School of Medicine, 1-1 Yanagido, Gifu 501-1194, Japan; 2 Department of Food and Culture science, Aichi Bunkyo Women's College, 2-9-17 Inaba, Inazawa, Aichi 492-8521, Japan

**Keywords:** blood pressure, child, preschool, diet records, seaweed, nutrition

## Abstract

**Background:**

Few studies have examined whether dietary factors might affect blood pressure in children. We purposed to investigate whether seaweed intake is associated with blood pressure level among Japanese preschool children.

**Methods:**

The design of the study was cross-sectional and it was conducted in autumn 2006. Subjects were healthy preschoolers aged 3-6 years in Aichi, Japan. Blood pressure and pulse were measured once by an automated sphygmomanometer, which uses oscillometric methods. Dietary data, including seaweed intake, were assessed using 3-day dietary records covering 2 consecutive weekdays and 1 weekend day. Of a total of 533 children, 459 (86.1 percent) agreed to be enrolled in our study. Finally, blood pressure measurement, complete dietary records and parent-reported height and weight were obtained for 223 boys and 194 girls.

**Results:**

When we examined Spearman's correlation coefficients, seaweed intake was significantly negatively related to systolic blood pressure in girls (*P *= 0.008). In the one-way analysis of covariance for blood pressure and pulse after adjustments for age and BMI, the boys with the lowest, middle and highest tertiles of seaweed intake had diastolic blood pressure readings of 62.8, 59.3 and 59.6 mmHg, respectively (*P *= 0.11, trend *P *= 0.038). Girls with higher seaweed intake had significantly lower systolic blood pressure readings (102.4, 99.2 and 96.9 mmHg for girls with the lowest, middle and highest tertiles of seaweed intake, respectively; *P *= 0.037, trend *P *= 0.030).

**Conclusion:**

Our study showed that seaweed intake was negatively related to diastolic blood pressure in boys and to systolic blood pressure in girls. This suggests that seaweed might have beneficial effects on blood pressure among children.

## Background

Hypertension, which often coexists with diabetes, dyslipidemia and obesity, promotes atherosclerosis and contributes to the development of cardiovascular disease [1]. Hypertension is generally unusual among children, but many studies have shown the tracking of blood pressure from childhood to adulthood [2], and some studies have indicated that the process of atherosclerosis starts in childhood [3,4]. Thus, early intervention for high blood pressure is important in order to prevent cardiovascular disease later in life.

Although there is much evidence for dietary risk factors for hypertension in adults [5-13], few studies of children have been reported [14-21]. Restricted salt intake and reduced alcohol consumption are recommended for adults [6]. The Dietary Approaches to Stop Hypertension (DASH diet), which is rich in fruits, vegetables and low-fat dairy products, has beneficial effects on blood pressure among adults [5-8]. The diet is rich in potassium and calcium, which have been reported to reduce blood pressure among adults [8,9,13]. A negative association between magnesium and blood pressure has also been reported among adults in some papers [10,11,13]. However, the role of diet in blood pressure among children is not well understood.

Seaweeds are traditional Japanese foods, and are consumed as they are (nori, kombu, hijiki) or as ingredients of rice balls (nori) and salads or soups (wakame). Seaweeds contain large quantities of dietary fiber, minerals, vitamins and polysaccharides [22]. Several experimental studies in animals [23-27] have shown that feeding on seaweed or its extract lowers blood pressure, suggesting that seaweed intake might affect blood pressure in humans. However, only a few epidemiological studies have reported an association between seaweed intake and blood pressure, and the results have been inconsistent [28-31]. These studies were conducted among adults, and the relationship of seaweed intake to blood pressure has not been investigated among children.

In this study, we purposed to investigate whether dietary seaweed intake is associated with blood pressure level among Japanese preschool children. We hypothesized that seaweed might beneficially affect blood pressure among children.

## Methods

### Subjects and Design

Subjects were children aged three to six years who attended one of two preschools in Aichi Prefecture, Japan. The details of the cohorts have been described elsewhere previously [32]. During October and November 2006, they underwent blood pressure measurement. Urine, which was first voided after a child waked up, was collected. Children's height, weight, health status, and lifestyles were inquired through a parent-administered questionnaire. Lifestyles included the time when they got up or went to bed, and a nap time. Physical activity was based on an outdoor playtime checklist by Burdette et al. [33]. The parents were also asked to record the children's dietary intake for covering 2 consecutive weekdays and 1 weekend day. Of a total of 533 preschool children, 459 (86.1 percent) agreed to be enrolled in our study, with their parents providing written informed consent. Finally, blood pressure measurement, complete dietary records and parent-reported height and weight were obtained for 417 of the children. This study protocol and the informed consent procedure were approved by the ethical board of Gifu University Graduate School of Medicine, Gifu, Japan.

### Dietary data

To collect nutritional data, diet including seaweed intake was assessed using 3-day dietary records covering 2 consecutive weekdays and 1 weekend day. The parents received written instructions on recording the food intakes of the children. According to the instructions, they recorded the amount and kind of foods, beverages and dishes which were consumed by their children during each of three days. When they were in trouble with recording, our stuffs assisted them on the phone. Because our subjects usually ate a school-provided lunch, we obtained the menus from each kindergarten and our staffs, which were dieticians, checked the quantity left over after each meal. Individual nutrient intake was estimated using the Japanese Standard Table of Food Composition, 5^th ^revised and enlarged edition [22]. We calculated energy intake in kilocalories per day and seaweed and salt intake in grams per day. Seaweed intake was converted to dry volume units.

### Blood pressure and other measurements

Systolic (SBP), diastolic blood pressure (DBP) and pulse were measured once by an automated sphygmomanometer (ES-H55, Terumo Co., Japan), which uses oscillometric methods. Measurements of blood pressure were conducted in midmorning. Children were not requested to be fasting. We used the appropriate size of blood pressure cuff based on each child's arm circumference. As a rule, measurements were taken from the upper arm. The subjects were measured in a sitting position after a few minutes of rest. The height and weight of children were based on parents' reports. Body mass index (BMI) was calculated as (weight in kg)/(height in m)^2^. From our subjects, we additionally obtained the measurements of the heights and weights of 103 of the children. Among them, intra-class correlation coefficients between measured and parent-reported data were 0.90, 0.96, and 0.78 in height, weight, and BMI, respectively.

### Statistical analysis

All analyses were performed separately for each sex. According to the nutrient density method, we divided seaweed and salt intake by total energy intake, and presented them as grams per 1,000 kcal of total energy. The dietary intakes such as total energy, seaweed and salt were skewed and hence were logarithmic transformed in all analyses.

The characteristics by sex were calculated as mean (standard deviation: SD). The geometric mean and 95% confidence interval were computed on the log-transformed values and converted back to the original scale of measurement. We used Spearman's correlation coefficients to detect the association of blood pressure and pulse with age, height, weight, BMI, and intakes of total energy, salt or seaweed.

We divided the subjects into three groups according to tertile category (low, middle or high) of seaweed intake. Tertiles were derived based on the distribution of seaweed consumption in the current population. In order to elucidate the relationships of seaweed intake with SBP, DBP and pulse, we used a one-way analysis of covariance (ANCOVA) after adjustments for age and BMI. Tests for linear trend were performed on multiple regression analyses using continues variables in seaweed intake.

All analyses were conducted using the SAS computer program, version 9.1 (SAS Institute). All P values were calculated by a two-sided test. A P value of less than 0.05 was considered statistically significant in all analyses.

## Results

The characteristics of studied subjects are shown in Table 1. Subjects were 223 boys and 194 girls. The averages (SD) of SBP and DBP were, respectively, 98.7 (13.0) and 60.6 (11.1) mmHg among boys. Those were 99.5 (12.4) mmHg for SBP and 62.2 (11.8) mmHg for DBP among girls. The average pulse rate of girls was higher than that of boys (93.6/min in boys and 96.5/min in girls: *P *= 0.047). Total energy intake was greater in boys than in girls (1446 kcal/day in boys and 1337 kcal/day in girls: *P *< 0.001). The geometric means of seaweed intake were 0.67 and 0.76 g/day in boys and girls, respectively (*P *= 0.096).

**Table 1:** Characteristics of 417 studied preschool children

	Boys		Girls	
n	223		194	
Age (years)^a^	5.14	(0.89)	5.12	(0.89)
Height (cm)^a^	106.9	(7.1)	106.6	(7.3)
Weight (kg)^a^	17.5	(2.6)	17.3	(2.6)
BMI (kg/m^2^)^a^	15.2	(1.3)	15.2	(1.5)
SBP (mmHg)^a^	98.7	(13.0)	99.5	(12.4)
DBP (mmHg)^a^	60.6	(11.1)	62.2	(11.8)
Pulse (beat/min)^a^	93.6	(15.4)	96.5	(13.6)
Diet				
Total energy intake (kcal/day)^b^	1446	(1411, 1483)	1337	(1304, 1372)
Salt intake (g/day)^b^	5.98	(5.77, 6.19)	5.74	(5.53, 5.96)
Salt intake (g/1000kcal)^b^	4.13	(4.02, 4.23)	4.28	(4.17, 4.40)
Seaweed intake (g/day)^b^	0.67	(0.58, 0.78)	0.76	(0.64, 0.89)
Seaweed intake (g/1000kcal)^b^	0.49	(0.42, 0.56)	0.58	(0.49, 0.68)

^a^mean (SD); ^b^geometrical mean and 95% confidence interval

SBP, systolic blood pressure; DBP, diastolic blood pressure; SD, standard deviation

Age, height and weight were significantly negatively correlated with pulse in boys and girls (Table 2). BMI was positively associated with SBP in boys and girls. Total energy intake was neither associated with SBP, DBP nor pulse. Salt intake was also neither associated with SBP, DBP nor pulse. Seaweed intake was weakly, but significantly negatively related to SBP in girls (Spearman's r = -0.190, P = 0.008). There were borderline-significant negative associations between seaweed intake and DBP in boys (Spearman's r = -0.112, P = 0.096) and girls (Spearman's r = -0.123, P = 0.089). In girls, the negative association between seaweed intake and pulse was also of borderline significance (Spearman's r = -0.137, P = 0.058).

**Table 2:** Spearman's correlation coefficients of blood pressure and pulse, with age, height, weight, BMI and diet

	Boys (n = 223)			Girls (n = 194)		
		
	SBP	DBP	pulse	SBP	DBP	pulse
Age	-0.125*	-0.016	-0.318***	-0.062	-0.045	-0.212***
Height	-0.046	-0.021	-0.275***	0.036	0.011	-0.247***
Weight	0.081	0.021	-0.261***	0.142**	0.089	-0.163**
BMI	0.155**	0.033	-0.012	0.187***	0.124*	0.120*
Total energy intake	0.090	0.001	-0.020	0.066	-0.041	-0.045
Salt intake^a^	0.018	0.005	-0.070	-0.097	-0.013	-0.058
Seaweed intake^a^	-0.071	-0.112*	-0.031	-0.190***	-0.123*	-0.137*

SBP, systolic blood pressure; DBP, diastolic blood pressure

^a^Each intake was controlled for total energy intake by the nutrient density method.

*P < 0.1. **P < 0.05. ***P < 0.01

Table 3 shows the estimated means of SBP, DBP and pulse according to the tertile category of dietary seaweed intake after adjustments for age and BMI. Boys with low, middle and high intake of seaweed had DBP readings of 62.8, 59.3 and 59.6 mmHg, respectively (*P *= 0.11, trend *P *= 0.038). Neither SBP nor pulse was associated with seaweed intake. Girls with higher seaweed intake had significantly lower SBP than those with lower seaweed intake (*P *= 0.037, trend *P *= 0.030). The DBP of girls with low seaweed intake was higher than those of middle and high intake of seaweed.

**Table 3:** Adjusted means of blood pressure and pulse according to the tertiles of dietary seaweed intake^a^

	Tertiles of seaweed intake^b^						*P*-value	*Liner trend P*
			
	low		middle		high			
Boys (n = 223)	75		74		74			
Median (range)	0.06 (0,0.15)		0.36 (0.16,0.68)		1.11 (0.71,3.11)			
SBP (mmHg)^c^	100.0	(97.1-103.0)	97.9	(94.9-100.8)	98.1	(95.1-101.0)	0.52	0.31
DBP (mmHg)^c^	62.8	(60.2-65.3)	59.3	(56.7-61.8)	59.6	(57.1-62.2)	0.11	0.038
Pulse (/min)^c^	93.4	(90.0-96.8)	94.2	(90.8-97.7)	93.3	(89.8-96.7)	0.91	0.89
Girls (n = 194)	65		65		64			
Median (range)	0.07 (0,0.22)		0.44 (0.22,0.76)		1.30 (0.77,7.98)			
SBP (mmHg)^c^	102.4	(99.4-105.4)	99.2	(96.2-102.2)	96.9	(93.9-99.9)	0.037	0.030
DBP (mmHg)^c^	65.1	(62.2-67.9)	60.4	(57.5-63.3)	61.2	(58.4-64.1)	0.055	0.25
Pulse (/min)^c^	98.8	(95.6-102.1)	96.3	(93.1-99.6)	94.2	(90.9-97.5)	0.15	0.092

SBP, systolic blood pressure; DBP, diastolic blood pressure

^a^Adjustments for age and BMI.

^b^Seaweed intake was controlled for total energy intake by the nutrient density method.

^c^Estimated mean and 95% confidence interval

Among seaweeds, nori (dried purple laver) was taken most common among our subjects (geometrical mean: 0.37 g/day). The association of nori intake with blood pressure was similar to that of total seaweed intake. For example, boys with low, middle and high intake of nori had DBP readings of 61.4, 61.7 and 58.6 mmHg, respectively (trend *P *= 0.057). Girls with low, middle and high intake of nori had SBP readings of 100.6, 101.1 and 97.0 mmHg, respectively (trend *P *= 0.040).

In order to see whether using parent-reported height and weight could influence the results, we assessed the association between seaweed intake and blood pressure among 53 boys and 50 girls whose measurements of height and weight were obtained. When the measured BMI was used as a confounder, boys with low, middle and high intake of seaweed had DBP readings of 64.7, 57.7 and 54.8 mmHg, respectively (trend *P *= 0.075). Girls with low, middle and high intake of seaweed had SBP readings of 99.6, 98.9 and 92.5 mmHg, respectively (trend *P *= 0.032).

We repeated the same analyses including other dietary factors as a confounder. Salt or sodium intake did not affect the negative association between seaweed intake and DBP among boys. The negative association between seaweed intake and SBP was also unaltered among girls. Additional adjustment for other mineral intakes (potassium, calcium, magnesium) also did not change the associations observed. When vegetable, fruit or fat intake was added as a confounder, the results were not substantially altered.

In addition, we assessed the association between seaweed intake and blood pressure after additional adjustment for non-dietary lifestyle factors. Sleeping habits (sleeping time in hours or the time when they got up or went to bed) or sedentary lifestyle time (total minutes of watching television or video-gaming, and reading a book) did not affect the association observed. We got data about physical activity only among 110 boys (Mean (SD): 98.0 (13.3) mmHg for SBP and 60.4 (12.1) mmHg for DBP) and 85 girls (Mean (SD): 98.5 (13.6) mmHg for SBP and 60.7 (SD: 10.2) mmHg for DBP). Although the observed association became non- significant after additional adjustment for physical activity, the tendency in the negative association between seaweed intake and blood pressure was not altered. After additional adjustments for passive smoking or years of mother's education, the negative association between seaweed intake and blood pressure were observed.

Finally, we re-examined the association between seaweed intake and blood pressure after excluding 4 children who took medications for common cold on the day of blood pressure measurement. Along with the results among all subjects, seaweed intake was negatively related to DBP in boys (trend *P *= 0.024) and to SBP in girls (trend *P *= 0.028).

## Discussion

Although there is much evidence for dietary risk factors for hypertension in adults, few studies of children have been reported [14-21]. Even the association between salt intake and blood pressure has been inconsistent among children [14,15]. Several studies have demonstrated the negative associations of blood pressure with calcium and magnesium among children [16-19], but very few researchers have examined whether the other dietary factors might affect blood pressure in childhood [19-21]. To our knowledge, this study is the first to demonstrate an association between seaweed intake and blood pressure in healthy children. Seaweed intake was negatively related to DBP in boys and to SBP in girls. Although we cannot prove a causal relationship because of a cross-sectional design of this study, the finding suggests that seaweed might have beneficial effects on blood pressure among children.

Our study of 3- to 6-year-old Japanese children showed that the difference between the highest and lowest tertiles was 3.5 mmHg in DBP among boys and 5.5 mmHg in SBP among girls. Decreasing blood pressure in healthy children would be potentially beneficial for blood pressure control in the future. Among Japanese adults aged 40 to 49 years, the multivariate-adjusted hazard ratio of all-cause mortality for each 10-mmHg SBP increase was reported to be 1.37 times in men and 1.19 in women during 9.8 years of follow-up [34]. That for each 10-mm Hg DBP was 1.46 times in men and 1.40 times in women.

The negative association between seaweed and blood pressure in our study is supported by the results of several experimental studies [23-27]. A diet containing powdered brown seaweed lowered blood pressure and reduced the incidence of stroke in salt-loaded, stroke-prone spontaneously hypertensive rats [SHRs] [23]. Hydrolysates of wakame (Undaria pinnatifida), a kind of seaweed, were reported to decrease systolic blood pressure after oral administration in SHRs [24]. Peptides isolated from wakame also had inhibitory activity for angiotensin 1-conversing enzyme, causing an antihypertensive effect [25-27]. In addition, Ikeda et al. found that the administration of wakame delayed the development of stroke signs and improved the survival rate of salt-loaded, stroke-prone SHRs, although there was no significant difference in blood pressure changes compared with the control group [35]. These findings suggest that seaweed may have preventive effects on hypertension and cerebrovascular diseases.

However, only a few epidemiological studies have reported an association between seaweed intake and blood pressure, and the results have been inconsistent [28-31]. Among 62 middle-aged patients with mild hypertension in Sweden, Krotkiewski et al. observed a significant decrease in mean blood pressure after the patients were given 12 and 24 g/day seaweed fiber for 4 weeks [28]. In hypertensive elderly Japanese patients, systolic and diastolic blood pressure decreased after the patients received daily doses of 5 g of dried seaweed powder for 8 weeks [29]. In a cross-sectional study of 190 hypertensive elderly Japanese patients, the patients treated with a low dose of a single drug ate more fruits and seaweed than the patients treated with a high dose of a single drug or multiple drugs, which suggested that the habitual intake of these food might help the control of blood pressure [30]. However, among 7,081 Korean men 30 years of age and older, participants with metabolic syndrome, including hypertension, showed a higher intake of seaweed and oily foods than did participants without metabolic syndrome [31]. These studies were conducted among adults, and, in this study, the relationship of seaweed intake to blood pressure has been first demonstrated among children.

In our study, seaweed seemed to have greater relationships with DBP than SBP among boys. Meanwhile, it seemed to have greater relationships with SBP than DBP among girls. Although the effects of seaweed intake on blood pressure might be different in mechanism between boys and girls, the reason for the discrepancy is unclear. In the studies among adults, one study [31] included only men and the others [28-30] analyzed the association in mixed group of men and women. More reports are needed to determine the association by sex.

The underlying mechanism responsible for the association between seaweed and blood pressure remains to be clarified. Seaweed contains large quantities of minerals and alginate, which is a kind of dietary fiber. Alginate has been reported to reduce blood pressure [36], and Yamori et al. presumed that alginic acid in seaweed may cause the inhibition of intentional sodium absorption [23]. Potassium, calcium and magnesium also have been reported to reduce blood pressure in observational studies [13,16,17] and intervention trials [9-11,18]. However, the results among our subjects did not show associations between dietary potassium, calcium and magnesium intake and blood pressure. Nonetheless, we cannot deny the possibility that simultaneous intake of several minerals through seaweed may be effective for blood pressure. Alternatively, other ingredients may play a role in the control of blood pressure since alginate or each mineral in seaweed is lower than the effective dose needed to lower blood pressure. The whole diet pattern including seaweed intake is also possible to be responsible for the lower blood pressure.

Although a dietary record would be more accurate or better if it had been used for a longer time or repeated over different seasons, using a dietary record was one of merits in this study. One limitation is that blood pressure was measured only once, which may have caused a large measurement error. However, it is unlikely that such a measurement error was directly dependent on seaweed intake. Nonetheless, repeated measurements are best practice and necessary in future studies. Another limitation was that height and weight were reported by the parents. However, the correlation coefficient between parents' reports and measured ones ranged from 0.90 to 0.96 for height and 0.95 to 0.99 for weight among 170 first-grade and 206 fourth-grade Japanese children [37]. In our supplementary analysis among 103 children, the intra-class correlation between parent-reported and measured height and weight was high. Furthermore, the negative associations between seaweed intake and blood pressure were observed among them after the measured BMI was used as a confounder. Therefore, these differences would not greatly change the associations observed in our study. We also must note that BMI might not be necessarily a very good maker of fatness in growing children. Finally, the generalizability of our study is limited by the fact that our subjects were ethnically homogeneous Japanese children, whose diets differ from those of Western children.

## Conclusions

We have demonstrated a negative association between dietary seaweed intake and blood pressure among healthy children. Seaweed is a popular traditional foodstuff that is widely eaten among both children and adults in Japan. Not only do our results suggest that seaweed intake may have beneficial effects on blood pressure in children, but they also provide the possibility of creating a new, earlier-in-life strategy for the prevention of hypertension in adults.

## Abbreviations

BMI: Body mass index; DBP: diastolic blood pressure; SBP: systolic blood pressure.

## Authors' contributions

Author KW wrote the paper. Author CN designed the study and directed its implementation, including quality assurance and control. Author KN helped designing the study's analytic strategy. Author YT, MT, and YS conduct the literature review and prepare the Discussion sections of the text. Author KW, SO, KY and KA helped supervise the field activities. All authors read and approved the final manuscript.

## Competing interests

The authors declare that they have no competing interests.

## References

[B1] Ueshima H Sekikawa A Miura K Turin TC Takashima N Kita Y Watanabe M Kadota A Okuda N Kadowaki T Cardiovascular disease and risk factors in Asia: a selected review Circulation 2008 118 2702 2709 10.1161/CIRCULATIONAHA.108.790048 19106393 PMC3096564

[B2] Chen X Wang Y Tracking of blood pressure from childhood to adulthood: a systematic review and meta-regression analysis Circulation 2008 117 3171 3180 10.1161/CIRCULATIONAHA.107.730366 18559702 PMC3568631

[B3] Berenson GS Srinivasan SR Bao W Newman WP Tracy RE Wattigney WA Association between multiple cardiovascular risk factors and atherosclerosis in children and young adults. The Bogalusa Heart Study N Engl J Med 1998 338 1650 1656 10.1056/NEJM199806043382302 9614255

[B4] Beauloye V Zech F Tran HT Clapuyt P Maes M Brichard SM Determinants of early atherosclerosis in obese children and adolescents J Clin Endocrinol Metab 2007 92 3025 3032 10.1210/jc.2007-0619 17519311

[B5] Sacks FM Svetkey LP Vollmer WM Appel LJ Bray GA Harsha D Obarzanek E Conlin PR Miller ER Simons-Morton DG Effects on blood pressure of reduced dietary sodium and the Dietary Approaches to Stop Hypertension (DASH) diet. DASH-Sodium Collaborative Research Group N Engl J Med 2001 344 3 10 10.1056/NEJM200101043440101 11136953

[B6] Chobanian AV Bakris GL Black HR Cushman WC Green LA Izzo JL Jr Jones DW Materson BJ Oparil S Wright JT Jr Roccella EJ Seventh report of the Joint National Committee on Prevention, Detection, Evaluation, and Treatment of High Blood Pressure Hypertension 2003 42 1206 1252 10.1161/01.HYP.0000107251.49515.c2 14656957

[B7] Vollmer WM Sacks FM Ard J Appel LJ Bray GA Simons-Morton DG Conlin PR Svetkey LP Erlinger TP Moore TJ Karanja N Effects of diet and sodium intake on blood pressure: subgroup analysis of the DASH-sodium trial Ann Intern Med 2001 135 1019 1028 11747380 10.7326/0003-4819-135-12-200112180-00005

[B8] Chobanian AV Hill M National Heart, Lung, and Blood Institute Workshop on Sodium and Blood Pressure: a critical review of current scientific evidence Hypertension 2000 35 858 863 10775551 10.1161/01.hyp.35.4.858

[B9] McCarron DA Morris CD Blood pressure response to oral calcium in persons with mild to moderate hypertension. A randomized, double-blind, placebo-controlled, crossover trial Ann Intern Med 1985 103 825 831 3904559 10.7326/0003-4819-103-6-825

[B10] Witteman JC Grobbee DE Derkx FH Bouillon R de Bruijn AM Hofman A Reduction of blood pressure with oral magnesium supplementation in women with mild to moderate hypertension Am J Clin Nutr 1994 60 129 135 8017327 10.1093/ajcn/60.1.129

[B11] Hatzistavri LS Sarafidis PA Georgianos PI Tziolas IM Aroditis CP Zebekakis PE Pikilidou MI Lasaridis AN Oral Magnesium Supplementation Reduces Ambulatory Blood Pressure in Patients With Mild Hypertension Am J Hypertens 2009 22 1070 1075 10.1038/ajh.2009.126 19617879

[B12] Zhou B Zhang X Zhu A Zhao L Zhu S Ruan L Zhu L Liang S The relationship of dietary animal protein and electrolytes to blood pressure: a study on three Chinese populations Int J Epidemiol 1994 23 716 722 10.1093/ije/23.4.716 8002184

[B13] Van Leer EM Seidell JC Kromhout D Dietary calcium, potassium, magnesium and blood pressure in the Netherlands Int J Epidemiol 1995 24 1117 1123 10.1093/ije/24.6.1117 8824852

[B14] Simons-Morton DG Obarzanek E Diet and blood pressure in children and adolescents Pediatr Nephrol 1997 11 244 249 10.1007/s004670050271 9090675

[B15] He FJ Marrero NM Macgregor GA Salt and blood pressure in children and adolescents J Hum Hypertens 2008 22 4 11 10.1038/sj.jhh.1002268 17823599

[B16] Gillman MW Oliveria SA Moore LL Ellison RC Inverse association of dietary calcium with systolic blood pressure in young children Jama 1992 267 2340 2343 10.1001/jama.267.17.2340 1564773

[B17] Papandreou D Stamou M Malindretos P Rousso I Mavromichalis I Prevalence of hypertension and association of dietary mineral intake with blood pressure in healthy schoolchildren from northern Greece aged 7-15 years Ann Nutr Metab 2007 51 471 476 10.1159/000111169 18025822

[B18] Gillman MW Hood MY Moore LL Nguyen US Singer MR Andon MB Effect of calcium supplementation on blood pressure in children J Pediatr 1995 127 186 192 10.1016/S0022-3476(95)70293-8 7636641

[B19] Simon JA Obarzanek E Daniels SR Frederick MM Dietary cation intake and blood pressure in black girls and white girls Am J Epidemiol 1994 139 130 140 8296780 10.1093/oxfordjournals.aje.a116975

[B20] Ulbak J Lauritzen L Hansen HS Michaelsen KF Diet and blood pressure in 2.5-y-old Danish children Am J Clin Nutr 2004 79 1095 1102 15159241 10.1093/ajcn/79.6.1095

[B21] Ayer JG Harmer JA Xuan W Toelle B Webb K Almqvist C Marks GB Celermajer DS Dietary supplementation with n-3 polyunsaturated fatty acids in early childhood: effects on blood pressure and arterial structure and function at age 8 y Am J Clin Nutr 2009 90 438 446 10.3945/ajcn.2009.27811 19515739

[B22] Science and Technology Agency Ed Standard Tables of Food Composition in Japan (5th revised and enlarged edition) (in Japanese) 2008 Tokyo Bureau of the Ministry of Finance

[B23] Yamori Y Nara Y Tsubouchi T Sogawa Y Ikeda K Horie R Dietary prevention of stroke and its mechanisms in stroke-prone spontaneously hypertensive rats--preventive effect of dietary fibre and palmitoleic acid J Hypertens Suppl 1986 4 S449 452 3023589

[B24] Sato M Oba T Yamaguchi T Nakano T Kahara T Funayama K Kobayashi A Nakano T Antihypertensive effects of hydrolysates of wakame (Undaria pinnatifida) and their angiotensin-I-converting enzyme inhibitory activity Ann Nutr Metab 2002 46 259 267 10.1159/000066495 12464726

[B25] Sato M Hosokawa T Yamaguchi T Nakano T Muramoto K Kahara T Funayama K Kobayashi A Nakano T Angiotensin I-converting enzyme inhibitory peptides derived from wakame (Undaria pinnatifida) and their antihypertensive effect in spontaneously hypertensive rats J Agric Food Chem 2002 50 6245 6252 10.1021/jf020482t 12358510

[B26] Suetsuna K Nakano T Identification of an antihypertensive peptide from peptic digest of wakame (Undaria pinnatifida) J Nutr Biochem 2000 11 450 454 10.1016/S0955-2863(00)00110-8 11091100

[B27] Suetsuna K Maekawa K Chen JR Antihypertensive effects of Undaria pinnatifida (wakame) peptide on blood pressure in spontaneously hypertensive rats J Nutr Biochem 2004 15 267 272 10.1016/j.jnutbio.2003.11.004 15135150

[B28] Krotkiewski M Aurell M Holm G Grimby G Szczepanik J Effects of a sodium-potassium ion-exchanging seaweed preparation in mild hypertension Am J Hypertens 1991 4 483 488 1873002 10.1093/ajh/4.6.483

[B29] Hata Y N K Clinical effects of brown seaweed, Undaria pinnatifida (wakame), on blood pressure in hypertensive subjects Journal of clinical biochemistry and nutrition 2001 30 43 53

[B30] Ono A Shibaoka M Yano J Asai Y Fujita T Eating habits and intensity of medication in elderly hypertensive outpatients Hypertens Res 2000 23 195 200 10.1291/hypres.23.195 10821126

[B31] Shin A Lim SY Sung J Shin HR Kim J Dietary intake, eating habits, and metabolic syndrome in Korean men J Am Diet Assoc 2009 109 633 640 10.1016/j.jada.2008.12.015 19328258

[B32] Wada K Nakamura K Masue T Sahashi Y Ando K Nagata C Soy intake and urinary sex hormone levels in preschool Japanese children Am J Epidemiol 2011 173 998 1003 10.1093/aje/kwr006 21427172

[B33] Burdette HL Whitaker RC Daniels SR Parental report of outdoor playtime as a measure of physical activity in preschool-aged children Arch Pediatr Adolesc Med 2004 158 353 357 10.1001/archpedi.158.4.353 15066875

[B34] Murakami Y Hozawa A Okamura T Ueshima H Relation of blood pressure and all-cause mortality in 180,000 Japanese participants: pooled analysis of 13 cohort studies Hypertension 2008 51 1483 1491 10.1161/HYPERTENSIONAHA.107.102459 18443234

[B35] Ikeda K Kitamura A Machida H Watanabe M Negishi H Hiraoka J Nakano T Effect of Undaria pinnatifida (Wakame) on the development of cerebrovascular diseases in stroke-prone spontaneously hypertensive rats Clin Exp Pharmacol Physiol 2003 30 44 48 10.1046/j.1440-1681.2003.03786.x 12542452

[B36] Chaki T Kajimoto N Ogawa H Baba T Hiura N Metabolism and calcium antagonism of sodium alginate oligosaccharides Biosci Biotechnol Biochem 2007 71 1819 1825 10.1271/bbb.60620 17690483

[B37] Sekine M Yamagami T Hamanishi S Kagamimori S Accuracy of the estimated prevalence of childhood obesity from height and weight values reported by parents: results of the Toyama Birth Cohort study J Epidemiol 2002 12 9 13 11848186 10.2188/jea.12.9 PMC10432257

